# Immersive augmented reality system for the training of pattern classification control with a myoelectric prosthesis

**DOI:** 10.1186/s12984-021-00822-6

**Published:** 2021-02-04

**Authors:** Alexander Boschmann, Dorothee Neuhaus, Sarah Vogt, Christian Kaltschmidt, Marco Platzner, Strahinja Dosen

**Affiliations:** 1 Computer Engineering Group, Department of Computer Science, Faculty of Computer Science, Electrical Engineering and Mathematics, Paderborn University, Paderborn, Germany; 2 Exercise Science & Neuroscience Unit, Department Exercise and Health, Faculty of Science, Paderborn University, Paderborn, Germany; 3 Department of Health Science and Technology, Faculty of Medicine, Aalborg University, Aalborg, Denmark

**Keywords:** Prosthesis control, Pattern classification, Myoelectric control, Force feedback, Augmented reality, Training

## Abstract

**Background:**

Hand amputation can have a truly debilitating impact on the life of the affected person. A multifunctional myoelectric prosthesis controlled using pattern classification can be used to restore some of the lost motor abilities. However, learning to control an advanced prosthesis can be a challenging task, but virtual and augmented reality (AR) provide means to create an engaging and motivating training.

**Methods:**

In this study, we present a novel training framework that integrates virtual elements within a real scene (AR) while allowing the view from the first-person perspective. The framework was evaluated in 13 able-bodied subjects and a limb-deficient person divided into intervention (IG) and control (CG) groups. The IG received training by performing simulated clothespin task and both groups conducted a pre- and posttest with a real prosthesis. When training with the AR, the subjects received visual feedback on the generated grasping force. The main outcome measure was the number of pins that were successfully transferred within 20 min (task duration), while the number of dropped and broken pins were also registered. The participants were asked to score the difficulty of the real task (posttest), fun-factor and motivation, as well as the utility of the feedback.

**Results:**

The performance (median/interquartile range) consistently increased during the training sessions (4/3 to 22/4). While the results were similar for the two groups in the pretest, the performance improved in the posttest only in IG. In addition, the subjects in IG transferred significantly more pins (28/10.5 versus 14.5/11), and dropped (1/2.5 versus 3.5/2) and broke (5/3.8 versus 14.5/9) significantly fewer pins in the posttest compared to CG. The participants in IG assigned (mean ± std) significantly lower scores to the difficulty compared to CG (5.2 ± 1.9 versus 7.1 ± 0.9), and they highly rated the fun factor (8.7 ± 1.3) and usefulness of feedback (8.5 ± 1.7).

**Conclusion:**

The results demonstrated that the proposed AR system allows for the transfer of skills from the simulated to the real task while providing a positive user experience. The present study demonstrates the effectiveness and flexibility of the proposed AR framework. Importantly, the developed system is open source and available for download and further development.

## Background

The loss of a limb is a traumatic event that leaves a person with substantial disability, thereby dramatically decreasing the quality of life. The human hands are essential tools for daily life activities, allowing stable grasping, dexterous manipulation, and haptic exploration of the environment as well as communication with other people [1]. To replace the amputated hand, the subjects can be equipped with a myoelectric prosthesis. A prosthesis is a system that aims at replacing a human limb both morphologically and functionally. Therefore, an ultimate prosthetic hand should mimic the shape, size and weight of its biological counterpart [2], and restore the lost motor and sensory functions [3]. The myoelectric prosthesis is controlled by recording the electrical activity of user forearm muscles (electromyography, EMG) to detect his/her motor intention and command the prosthesis accordingly [4]. Most commercial systems are controlled using a two-channel direct and proportional control, which is effective and intuitive for a simple prosthesis (e.g., single degree of freedom gripper) [4]. For more advanced devices with several functions, machine learning can be used to extract multiple commands from the multichannel EMG [5], and indeed some solutions based on pattern classification are already commercially available (e.g., COAPT engineering [6] and MyoPlus from Otto Bock [7]).

Regardless of the method used, controlling a prosthesis is a challenging task for a prospective user [8]. They need to be able to activate muscles selectively as well as to modulate their activation for proportional control. When using a controller based on pattern recognition, the user needs to generate distinctive muscle activation patterns for each prosthesis function so that they can be recognized by a classifier. Therefore, an amputee requires training to master the myoelectric control [9]. Different training systems have been presented in the past, from simple visualization of myoelectric signals [10] to using these signals to play computer games [11–13] or control a virtual prosthesis shown on the screen [14–18].

To create an immersive training, the prosthesis control task can be implemented within a virtual reality environment. In [19], the residual limb was kinematically tracked to allow for visualizing the subject avatar with a prosthesis attached to the limb. The physics was also simulated, and a virtual Box and Block test was implemented and assessed in three able-bodied subjects. A recent study presented a virtual reality framework for interactive training and assessment of pattern classification myoelectric control integrating the target achievement control test [16, 17] and serious gaming (controlling a virtual crossbow) [20]. Although virtual reality systems can be effective instruments for myoelectric control training, they also have drawbacks. They require the user to wear a head-mounted display completely occluding the natural view of the surroundings and they can induce motion sickness [21].

Augmented reality (AR) is an emerging technology that not only overcomes some of these shortcomings but also creates a new experience of interaction for the user. In an AR system, the real-world view is combined with computer-generated information ranging from textual representation to animated virtual objects. The viewpoint used in an immersive environment impacts the subject’s sense of presence and embodiment. As shown in [22], while the third-person perspective is beneficial to the user’s general space awareness, the first-person perspective is the superior condition to induce a sense of embodiment and is more suitable for high-precision interaction with virtual objects, both of which are desired features in myoelectric rehabilitation systems.

Most of AR rehabilitation systems published between 2010 and 2016 used the third-person perspective, presumably also due to technical limitations in early head-mounted displays. These systems typically displayed a mirrored image of the user on a PC screen. In [23], an AR myoelectric training system capable of controlling a virtual arm blended into a mirrored third-person perspective image of the user in real-time. The system used a printed 2D marker attached to the subject’s body so that the virtual limb could move along with the user on the PC screen. A similar system endowed with gaming elements was presented in [24], in which the subjects used myoelectric control to shoot bullets from the virtual limb at spaceships sporadically appearing as overlays at different locations on a PC screen. The marker-based approach was further refined in [25] by adding more detailed and realistic virtual arm models and animations, and the system was applied as a treatment for phantom limb pain [26]. Instead of using printed markers as a visual anchor for the position and alignment of the virtual limb, the system presented in [27] relied on Microsoft Kinect as external motion-sensing hardware to dynamically connect the virtual limb to the user’s body.

As head-mounted displays with improved displaying hardware (e.g., built-in stereoscopic cameras and embedded processing) became more available, an increasing number of AR rehabilitation systems using the first-person perspective was presented. A myoelectric prosthesis-training simulator displaying a virtual prosthesis and virtual objects from the user’s view was shown in [28]. The system relied on two independent 2D-markers to connect the virtual limb and a virtual object to the real-world video stream. However, the system did not provide 3D experience since it relied on a mono-camera. In [29], a wireless kinematic tracking framework based on inertial measurement units was introduced to accurately predict trajectories of a real or prosthetic arm and translate it into an AR environment displayed by a Microsoft HoloLens. This system was extended in [30] by adding EMG-based control through the mapping of pre-defined gestures recognized by the Myo armband to the movements of a virtual prosthesis. The system also visualized forces that the virtual prosthesis applied to virtual objects on an external PC screen outside the AR environment. An AR prosthesis training system based on a Microsoft HoloLens for AR and Myo armband for EMG control was described in [31, 32]. The setup included an additional arm tracking system based on multiple inertial measurement units (IMUs) on the forearm, upper arm and chest for accurate arm trajectory estimation as well as a basic vibrotactile feedback system to indicate contact/no contact with virtual objects during reach and grasp tasks. A system based on the same AR and EMG setup was presented in [33]. However, instead of using external arm tracking hardware, the system relied on the built-in single IMU in the Myo armband to approximate the virtual limb position and orientation.

As mentioned before, the virtual training has several potential advantages. However, if and how much the skills acquired in the simulated environment can be transferred to the real task is still an open question [11, 13]. In the present study, we describe and demonstrate a novel system based on AR from the first-person perspective. The subject observed the real environment in front of him/her while a virtual prosthesis was attached to the limb (virtual reconstruction). They then used the prosthesis to interact with virtual objects placed within the real environment, where the interaction was governed by a simple physics simulation. A preliminary version of the system was presented at a conference [34]. The system described in this study represents a significant further development: in particular, multiple AR markers, interaction with multiple virtual objects and optional control of a real prosthesis are now supported. More realistic 3D models, animations and general performance improvements create a more immersive and realistic user experience. We have tested the system by implementing virtual prosthesis training using the clothespin test and assessed the transfer of learning to control a real prosthesis using a pre- and posttest protocol.

## Methods

### System architecture and operation

**Fig. 1:**
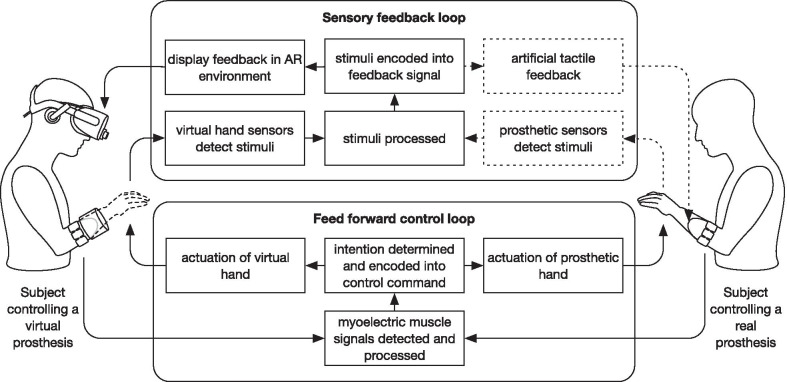
The AR system with closed-loop control. Pattern classification is applied on a multichannel EMG recorded using Myo armband to estimate user motion intention and command a virtual (left) or real prosthesis (right). The virtual prosthesis is sensorized and the data from the sensors are acquired and presented to the subject as visual feedback (e.g., a graphical bar embedded within a real scene).The functions shown as dotted boxes have not been implemented in the presented system, but they can be added subsequently for corresponding hardware via existing interfaces

The developed system uses a binocular head-mounted display (HMD) for real-time rendering of a virtual hand displayed as an augmented reality overlay extending from the residual limb of an amputee subject. The system implements closed-loop control of the virtual prosthesis (see Fig. 1). In the feedforward control loop, the system continuously acquires myoelectric signals from a wireless Myo armband EMG sensor (Thalmic Labs, US). From these signals, the intended movement and contraction force are determined by a pattern recognition subsystem and encoded into control commands for the prosthesis, as described below. In the sensory feedback loop, the system reads sensor data from the virtual arm (e.g. force applied to virtual objects) and encodes them into a feedback signal. The system can display the corresponding feedback to the user as an overlay in the AR environment. Importantly, the control loop was implemented so that it can also accommodate a real prosthetic hand (Michelangelo hand from Otto Bock).

We used an Ovrvision Pro [35] off-the-shelf stereovision camera to capture a video stream at 60 frames per second in 960 × 950 pixels resolution. Its viewing angle of 100° horizontal and 98° vertical is similar to the human field of view and provides a realistic quasi-orthoscopic view to the user. The cameras were mounted on the anterior side of the HMD and they, therefore, recorded the environment from the perspective of the user. The video stream was processed as described below and then projected to the HMD. Hence, the user was looking into the real scene in front of him into which virtual 3D objects were projected (AR).

Two custom AR markers suitable for standard scale-invariant feature detectors (e.g. SIFT [36]) in the user's field of view were utilized by our system: the arm marker that was placed on the residual limb could be freely moved inside the field of view while the scene marker remained at a fixed position (e.g., table surface). To achieve an optimal detection quality from various viewing angles resulting from different arm positions, a conical shape was chosen for the arm marker. The marker was placed around the forearm (residual limb) so that it could be detected even under forearm rotations. For the scene marker, a simple planar design was selected because it was only viewed from the front side. Both markers were created using high-contrast polygon patterns to provide a stable recognition performance. The positions detected by the marker-tracking module were used to integrate a virtual arm model and other virtual objects into the stereoscopic video data projected onto an Oculus Rift CV1 HMD. For EMG data acquisition, a wireless Myo armband was used to capture 8 bipolar channels of surface EMG at a sampling rate of 200 Hz. The virtual arm was controlled by the subject in real-time using a well-established EMG pattern recognition scheme, as explained below.

**Fig. 2:**
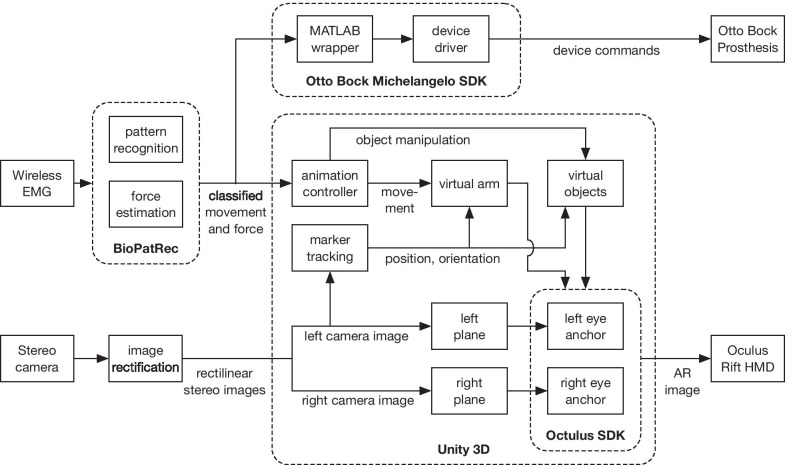
Overall system architecture with main components and data flow: BioPatRec was used for classification, marker tracking located the Arm and scene markers, and animation controller displayed the model of the arm (11 different movements) as well as virtual objects placed in the scene. The virtual models were integrated into the real scene recorded by the stereo camera and the final images were projected to the HMD. The details are explained in the text

A detailed scheme of the system architecture is shown in Fig. 2. For controlling either the virtual arm model or the real prosthesis, our system used a well-established EMG pattern recognition approach consisting of extraction of time-domain features (mean absolute value, waveform length, slope sign changes, and zero crossings) from the raw EMG signals and classification using linear discriminant analysis (LDA) [37]. In addition, the contraction strength was also determined by an envelope function. These steps were performed using BioPatRec [38], which is an open-source framework for pattern recognition control running in MATLAB (MathWorks, US). The classifier decision and contraction force were sent over TCP/IP to either Otto Bock Michelangelo software development kit (SDK) or to the animation controller module inside a Unity 3D application, which is described below. The window length for feature extraction and command generation through classification was set to 150 ms with an overlap of 50 ms.

The main component of the proposed system was an application developed using C# and Unity 3D, a state-of-the-art game engine and development environment. Each frame of the stereoscopic camera video stream was read from the device driver by a server application where it was decoded and processed. The left and right camera images were extracted and rectified to compensate for the barrel distortion from the wide-angle lenses using a pre-computed calibration matrix. The resulting rectilinear stereo images were then written into a shared memory location monitored by two DirectShow filters that transmitted the images to Unity 3D as two virtual webcam streams. Unity 3D rendered the images to the left and right plane in the virtual scene, providing the live real-world background scene to which the virtual objects were placed. For performance reasons, only the left camera image was used as input for the marker-tracking module. Here, we relied on Vuforia, a Unity 3D plugin, and SDK implementing a reliable state-of-the-art marker detection. When the AR arm and scene markers were detected, their positions and orientations were applied to the virtual arm model and the virtual scene. The arm model was a boned and rigged model of a realistic human arm created in Autodesk 3ds Max.

**Fig. 3:**
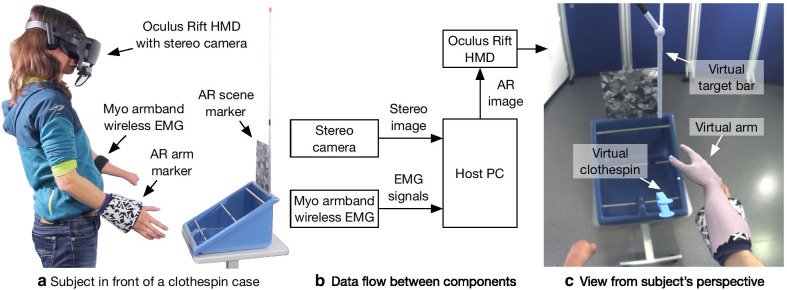
Experimental setup for the AR intervention showing a test subject with dysmelia controlling the virtual arm in an AR scene. The left **a** shows the scene including the system components mounted on the subject and a clothespin case positioned in front of the subject. The subject controlled the virtual hand using EMG signals that were recorded from the residual limb. The middle **b** reveals the connection and dataflow between the components and the host PC. The panel on the right **c** shows the view from the subject perspective with the virtual objects (hand, clothespin, and vertical bar) embedded in the real scene

We have implemented 11 realistic hand animations that can be applied to the arm model using cascaded blend trees method in Unity 3D. Available animations are open/palmar grasp, wrist pronation/supination, wrist extension/flexion, ulnar/radial deviation, lateral grasp, pinch grasp, and extension of the index finger. For the AR intervention phase of the experiment described in the next section, we used five of the implemented animations: hand open, palmar grasp, lateral grasp, and wrist pronation/supination. These animations corresponded to the movement classes (prosthesis commands) estimated by the LDA classifier. The speed of the virtual arm movement was proportional to the average level of muscle activity across the EMG channels normalized to the maximum activation. In addition to the virtual arm model, virtual objects such as clothespins, grip force indicators (feedback to the user) and handles for the clothespin test were dynamically created and rendered into the scene relative to the AR scene marker position (see section “Experimental assessment”). Finally, the animations were applied to the virtual arm model that was then passed along with the left and right plane objects as input to the Oculus Rift SDK. Here, the specific Oculus Rift lens distortion was applied to the resulting image, which was displayed inside the Oculus Rift. Figure 3 shows the complete system used by a test subject with dysmelia while she controlled the virtual arm in an AR scene. It should be noted that during the experiments the Myo armband EMG sensor was placed on the residual limb (left) while the AR arm marker was on the other arm (right) as shown in the figure. Consequently, the subject controlled the virtual right arm with the EMG signals of the residual limb (left). While the EMG sensor and the AR arm marker could be positioned on the same arm, it was not possible to place the EMG sensor under the prosthesis splint described in section “Experimental assessment”. Therefore, to make the control of the virtual prosthesis consistent with the real prosthesis, we placed the EMG sensor on the left and the real or virtual prosthesis on the right in all test subjects. This circumstance does not represent an intrinsic limitation of our system per se, since the AR arm marker can be placed on both arms and the system includes both a left and a right virtual arm that can be fully controlled. All test subjects reported that after a short period of familiarization they had no difficulties with this technical limitation. An example of what the system looks like with the Myo armband and the AR arm marker on the same arm is shown in Fig. 11.

### Experimental assessment

#### Participants

Thirteen able-bodied adults voluntarily participated in the experiment. They were separated into an AR intervention group (IG) (N = 7; 4 females; 3 males; 27.9 ± 3.9 years) and a control group (CG) (N = 6 males; 26.2 ± 5.7 years). The subjects were randomly assigned to the two groups. The participant with dysmelia followed the same protocol as the subjects in the intervention group, but her results were separately analysed as a case study (female, 33 yrs.). All subjects were naive to the purpose of the experiment, which has been approved by the Paderborn University ethical committee. Before being tested, each participant gave his or her written informed consent. The experiment was conducted in the laboratory of the computer engineering group at Paderborn University.

#### Experimental procedure

**Fig. 4:**
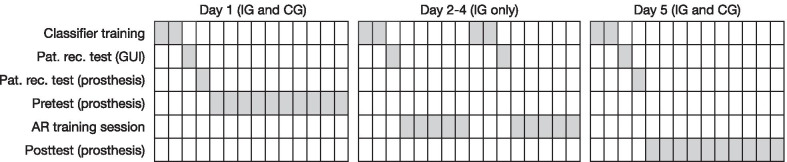
The experimental timeline. The initial assessment was conducted on Day 1 (pretest) for both the control and intervention groups. The intervention group received three days of training. The final assessment was performed on Day 5 for both groups. Each box in the timeline corresponds to approx. two minutes. IG and CG stand for the intervention and control group, respectively

The novel AR system was employed as an instrument for the training of myoelectric prosthesis control. The training was based on a virtual clothespin task, and the effects were evaluated using a pre-posttest experimental design. Between the tests, the intervention group completed 6 training sessions on 3 consecutive days using the described AR system. The control group completed pre- and posttest without any training in between. After the posttest, all participants answered a post questionnaire to obtain additional information about the subjective experience of the AR-supported training. The subjects were asked to rate (0–10) the level of difficulty of the real task with the real prosthesis, fun factor and motivation when using the AR system, and the perceived utility of the visual force feedback provided in AR. Figure 4 shows the timeline of the experimental procedure.

#### Pre- and Posttest Procedure: Clothespin test with a real prosthesis

Each participant completed a pre- and posttest that lasted approximately 1 h. For this test, a real prosthesis (Michelangelo hand from Otto Bock) was used. The prosthesis implements two grasp types (palmar and lateral) and wrist rotation (pronation/supination). The Myo armband was placed on the left arm and the prosthesis was mounted on the right arm using a custom-made splint. The position of the Myo armband and the prosthesis were documented to ensure a similar attachment of the devices in the posttest. The individual height of the test desk was also documented to ensure a similar experimental setup for the following training sessions (IG) and the posttest. The casing of the clothespin test was positioned on the desk. Importantly, the pins were modified by adding an LED and a switch so that when the pin handles contacted each other (pin fully open) the switch was closed. This activated the LED indicating that the subject “broke” the pin (so-called sensorized clothespin test [39]).

The pretest started with an EMG pattern recognition approach using BioPatRec [38]. Five different movements (open, lateral grasp, palmar grasp, pronation, and supination) were recorded 6 times for 5 s respectively with rest in between. A test of the EMG pattern recognition using BioPatRec was carried out afterward. If the subjects were able to activate the classes reliably (subjective assessment by the experimenter), they proceeded to control the prosthesis, otherwise, the training was repeated.

The participants were then familiarized with the experimental task. They were asked to grasp and move a clothespin using the prosthesis as well as to intentionally produce too much (LED activated) or too little force (pin slipped from the grasp) to understand all aspects of the task. If the control performance was not satisfactory, as subjectively assessed by the experimenter, the training was repeated for a maximum of 3 times.

**Fig. 5:**
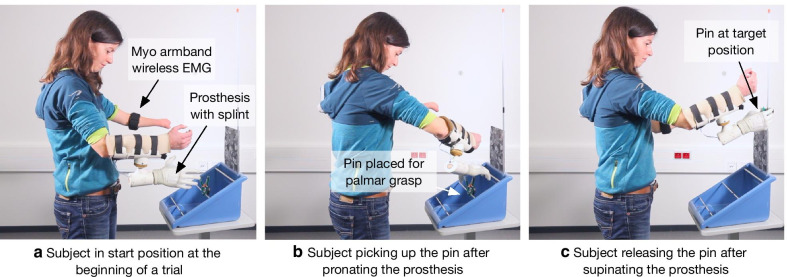
Experimental setup for the pre- and posttest procedure showing a subject with dysmelia controlling the real prosthesis. The clothespin case was positioned in front of the subject and the real prosthesis was mounted on a socket adapter on the sound arm and controlled using EMG signals that were recorded from the residual limb. The left **a** shows the subject in start position at the beginning of a trial, the prosthetic hand was open and perpendicular to the ground. The middle **b** depicts the subject picking up the pin placed on the middle horizontal bar using the palmar grasp after the prosthesis was pronated. The right **c** shows the subject releasing the pin at the target position using the hand open gesture after the prosthesis was supinated

Before starting the test, demonstrational videos for the lateral and palmar grasp were presented two times with additional instructions from the experimenter to explain the difference between the two grasp types. Following this, the test started. The task for the subjects was to grasp a pin attached to a horizontal bar, remove it, and relocate it to a vertical bar (Fig. 5). To achieve this, the subjects needed to close the hand and squeeze the pin, rotate the wrist to align the pin with the vertical bar, and open the hand to release the pin. Since the pins were sensorized, the subjects needed to apply the grasping force that was high enough to open the pin but lower than the pin “breaking” threshold. The test included pins with varying stiffness, which was indicated by the pin color (yellow, red, green, and blue). The minimal and maximal allowed forces and apertures for the pins are summarized in Table 1. The experimenter provided instructions and feedback until two clothespins were relocated correctly. The subject was instructed to alternate between palmar and lateral grasp across trials. In order to assess the subject’s ability to apply the right amount of grasping force, the task in the pre- and posttest required a strict control of this parameter. To prevent a subject from simply using the maximal force to accomplish the task we decided to treat each “broken” pin as a dropped pin in the conventional, non-sensorized clothespin relocation test. Therefore, if the subject activated the LED using an excessive amount of grip force or dropped the pin, the trial was deemed unsuccessful and the reallocation task was restarted. If the pin was “broken” for the second time, the subject continued with the next pin. The goal was to relocate as many pins as possible within 20 min (task duration). The number of successfully relocated, dropped, and “broken” pins were documented.

**Table 1:** Summary of minimal and maximal allowed forces and apertures for sensorized clothespins used

Pin color	Min aperture (%)	Max aperture (%)	Aperture window size (%)	Min force (%)	Max force (%)	Force window size (%)
Yellow	33	71	38	7	15	8
Red	33	66	33	13	23	10
Green	33	57	24	23	32	9
Blue	33	57	24	29	37	8

The force and aperture values are given relative to the prosthesis maximal grip force (100 N) and the clothespins maximal aperture (3.2 cm)

#### Augmented reality training: virtual clothespin task

The intervention group performed training using a simulated clothespin relocation task. The setup was similar to that used in the pre- and posttest. First, the Myo armband was placed on the left arm and the AR arm marker was placed on the right arm of the participant. The Myo armband was not repositioned between individual sessions on the same day. After this, the same EMG pattern recognition training as in the pre- and posttest sessions was conducted.

Every intervention session consisted of two AR training blocks that lasted 10 min each, with a break of approximately 5 min between the blocks. At the beginning of the first intervention session, instructional videos for the AR training were presented on a computer screen. Following this, the HMD was placed on the subject, and they practiced using the system for several minutes to get familiar with the AR environment. The experimenter provided instructions until the subject accomplished the first two trials successfully.

The subject used a virtual prosthesis to relocate virtual pins from the horizontal bar (real) to the vertical bar (virtual). The virtual pins used the same color-coding and simulated the same stiffness as the real pins. Additionally, the subjects received virtual feedback on the generated grasping force. The feedback was integrated into the real scene by showing a horizontal force bar near the virtual hand. The force bar displayed the generated grasping force as well as the target window that the subject should reach to grasp the pin successfully. If the applied force was above the target window, the virtual pin “exploded”. If the force dropped below the window while the pin was being relocated, the pin slipped from the grasp and “exploded”. The virtual vertical bar to which the pin should be relocated was rotated to different positions across trials. The subject was instructed to pronate or supinate the virtual hand to align the clothespins correctly to the bar. In summary, the virtual training was designed to motivate the subject to practice hand opening and closing, force modulation and wrist rotation.

**Fig. 6:**
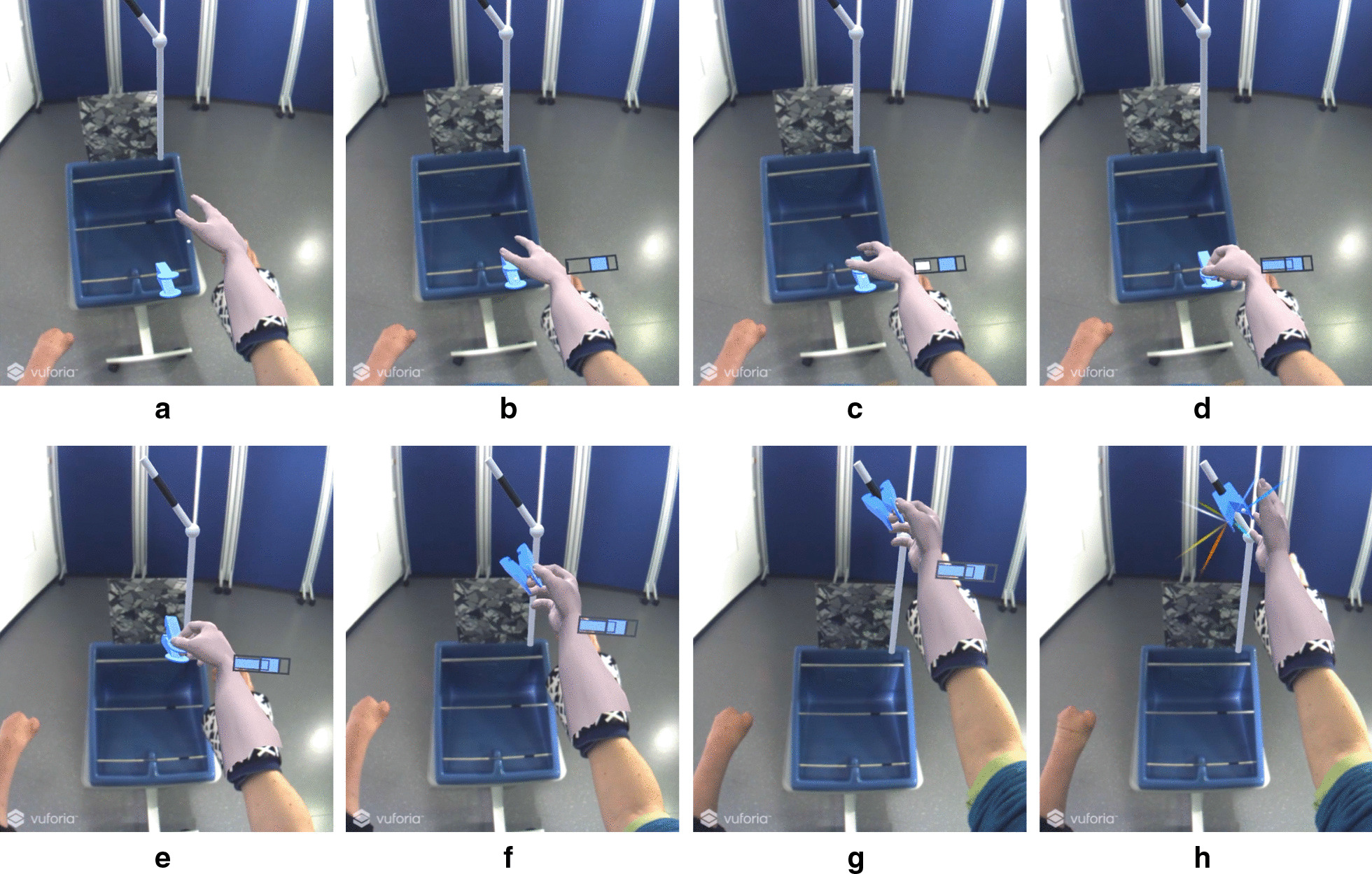
Snapshots from a successful trial showing the subject with dysmelia relocating a pin from a horizontal to the vertical bar by controlling a virtual hand using pattern classification. The snapshots (**a**) to (**h**) correspond to what the subject has seen looking through the head-mounted display, and the scene comprises both real (e.g., clothespin frame, horizontal bars) and virtual (e.g., hand, pin, vertical bar) objects, hence augmented reality. Note that the vertical bar is tilted and the black segment of the bar represents the target area where the pin should be attached. **b** to **g** show visual feedback on the grasping force displaying the generated force as well as the target range to grasp the pin successfully. The last panel (**h**) depicts a fireworks animation indicating a successful executing of the task

Figure 6 illustrates a trial in which a subject successfully relocated a virtual clothespin from the lower horizontal bar to the target position on the vertical bar. The figure shows the scene as perceived by the subject during the training. The subject approached the clothespin with the virtual hand (Fig. 6a). When the hand was close enough to grasp the virtual clothespin, the horizontal force bar appeared next to the hand, indicating the target force range required to grasp the pin successfully (i.e., without breaking it) (Fig. 6b). After the subject closed the hand using the selected grasp type and contacted the pin, the force produced by the hand was displayed as a moving bar. As long as the force was smaller than the target force range (Fig. 6c), the bar was shown in white color. When the generated force entered the target window, the color of the bar changed to the color of the clothespin and the clothespin could be detached from the horizontal bar and transported to the vertical bar (Fig. 6e and f). During the transport, the force feedback remained displayed so that the subject could control force to avoid breaking or dropping the pin. The subject rotated the virtual hand to align the pin to the vertical bar (Fig. 6g). Finally, they relaxed the muscles to release the clothespin and attach it to the designated segment of the bar (black area). To indicate a successful accomplishment of the task, a motivating fireworks animation was played (Fig. 6h). A video demonstrating the system components as well as performed tests is available on the project website (see "Supplementary information").

#### Data analysis

Statistical analysis was performed using MATLAB 2017b. The main outcome measure was the number of pins that the subjects successfully transferred within the fixed duration of the pre- and posttest (20 min). The number of failed pin transfers, when the subjects dropped or broke the pins, was also reported. Due to small sample sizes, non-parametric tests were used. The results of IG across sessions were compared using the Friedman test. A posthoc analysis was performed using Tukey’s honestly significant difference criterion for pairwise comparison. To compare the performance between pre- and posttest within the same group (IG and CG), the Wilcoxon signed-rank test was employed. To compare the performance between the groups (IG versus CG) in the same assessment (pre or post), the Mann–Whitney test was used. The results of the post questionnaire were normally distributed, and therefore, they were compared using a t-test for independent samples. The threshold of statistical significance was set at *p* < 0.05. The results are reported as median/interquartile range.

## Results

**Fig. 7:**
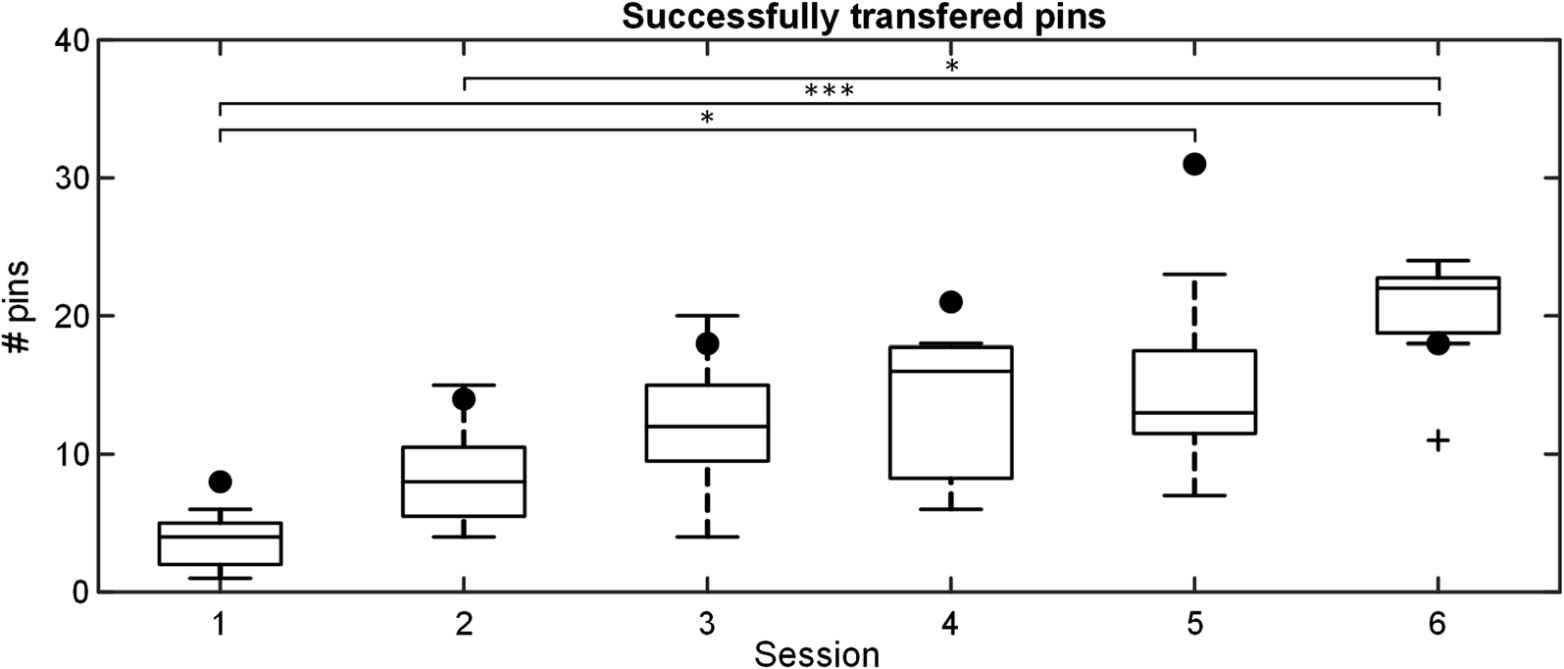
The number of successfully transferred pins across sessions for the subjects in the intervention group (boxplots) and the participant with dysmelia (black dots). The boxplots depict median (horizontal line), interquartile range (box), min and max values (whiskers), and outliers (cross). The horizontal bars indicate statistically significant difference (*, *p* < 0.05; ***, *p* < 0.001). Note that the performance consistently increased across the training sessions

The summary performance across the training sessions for the subjects in the IG is shown in Fig. 7. During the training, the subjects in IG performed virtual prosthesis control and clothespin task using the developed AR system. The difference in the number of successfully transferred pins across sessions was statistically significant (*p* < 0.001). The performance increased significantly between session 1 (4/3) and sessions 5 (13/6) and 6 (22/4), and between sessions 2 (8/5) and 6 (22/4). At the end of the training, the median number of successfully transferred pins increased more than fivefold compared to that in the first training session. Importantly, the limb deficient subject (black dots) demonstrated similar improvement in performance, and in fact, her score was among the best in most of the sessions.

**Fig. 8:**
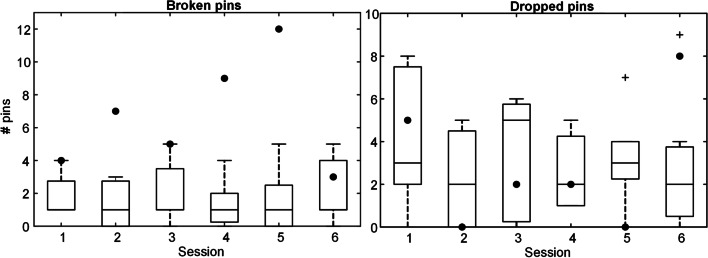
The number of pins that were broken (left) and dropped (right) by the subjects of the intervention group (boxplots) and the participant with dysmelia (black dots) across the training sessions. The boxplots depict median (horizontal line), interquartile range (box), min and max values (whiskers), and outliers (cross). The sum of broken and dropped pins corresponds to the total number of failed trials

Figure 8 shows the distribution of the failed trials across the training sessions for the subjects in the intervention group. There was no significant difference in the number of dropped or broken pins across the sessions. It seems that the subject with dysmelia had difficulties controlling the upper level of grasping force since she broke more pins compared to other participants, particularly in sessions 4 and 5.

**Fig. 9:**
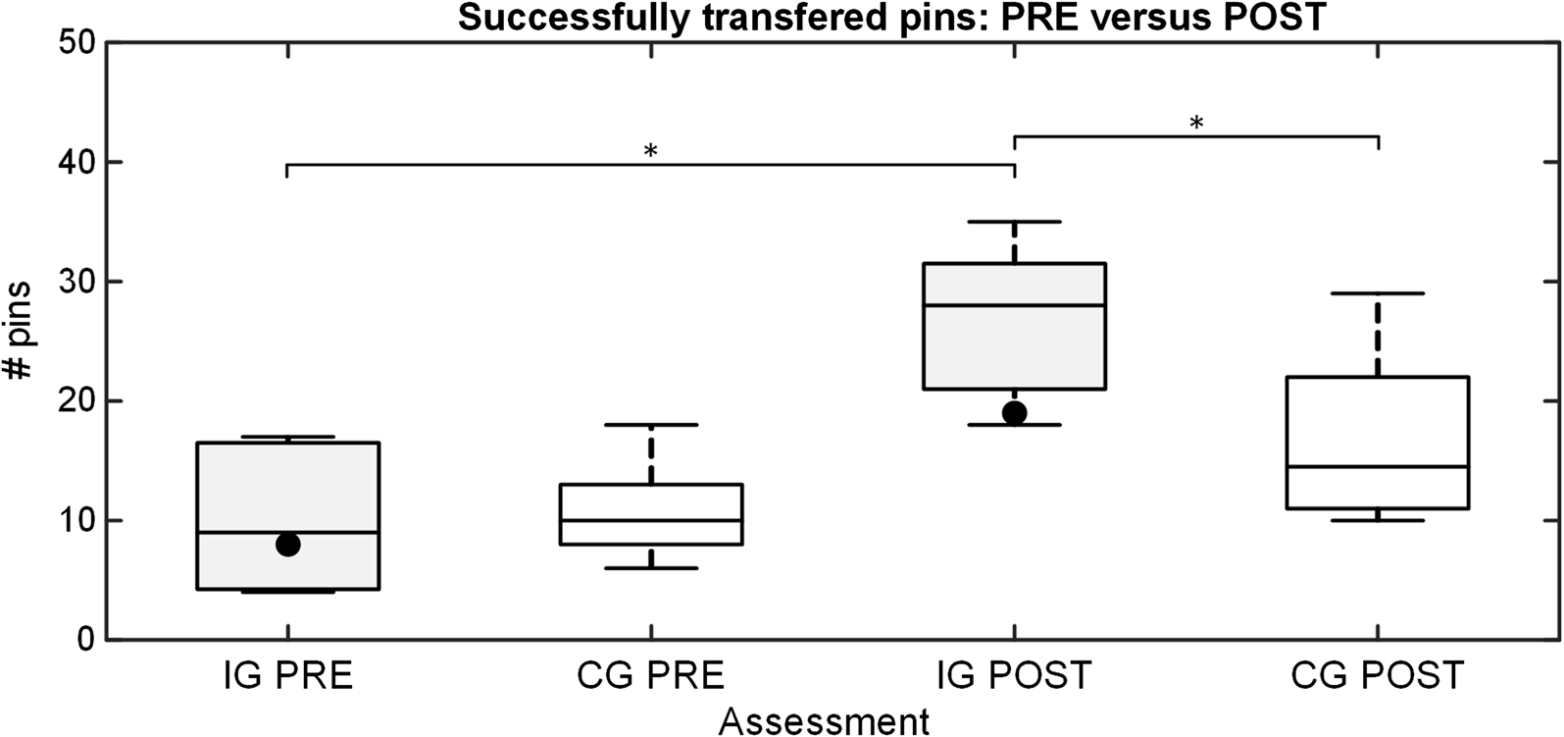
The number of successfully transferred pins for intervention and control groups (boxplots), and the subject with dysmelia (black dots) in pre- and posttest with the real prosthesis. The boxplots depict median (horizontal line), interquartile range (box), min and max values (whiskers), and outliers (cross). The horizontal bars indicate statistically significant difference (*, *p* < 0.05). IG and CG stand for intervention and control group, while PRE and POST refer to pre- and posttest, respectively. The performance of the intervention group increased significantly after the training, outperforming the control group in the posttest

The summary performance for the IG and CG, and the participant with dysmelia performing real-life clothespin task using Michelangelo prosthesis in pre- and posttest is shown in Fig. 9. During the period between pre- and posttest, the intervention group and the subject with dysmelia received training using the AR system. The performance of both groups was similar during the first test (pre), and it improved significantly (*p* < 0.05) from pre- to posttest only in the IG. In the posttest, the IG substantially outperformed (*p* < 0.05) the control group (28/10.5 versus 14.5/11). Importantly, the limb-deficient subject also transferred more pins after the training, from 8 in pre- to 19 in the posttest.

**Fig. 10:**
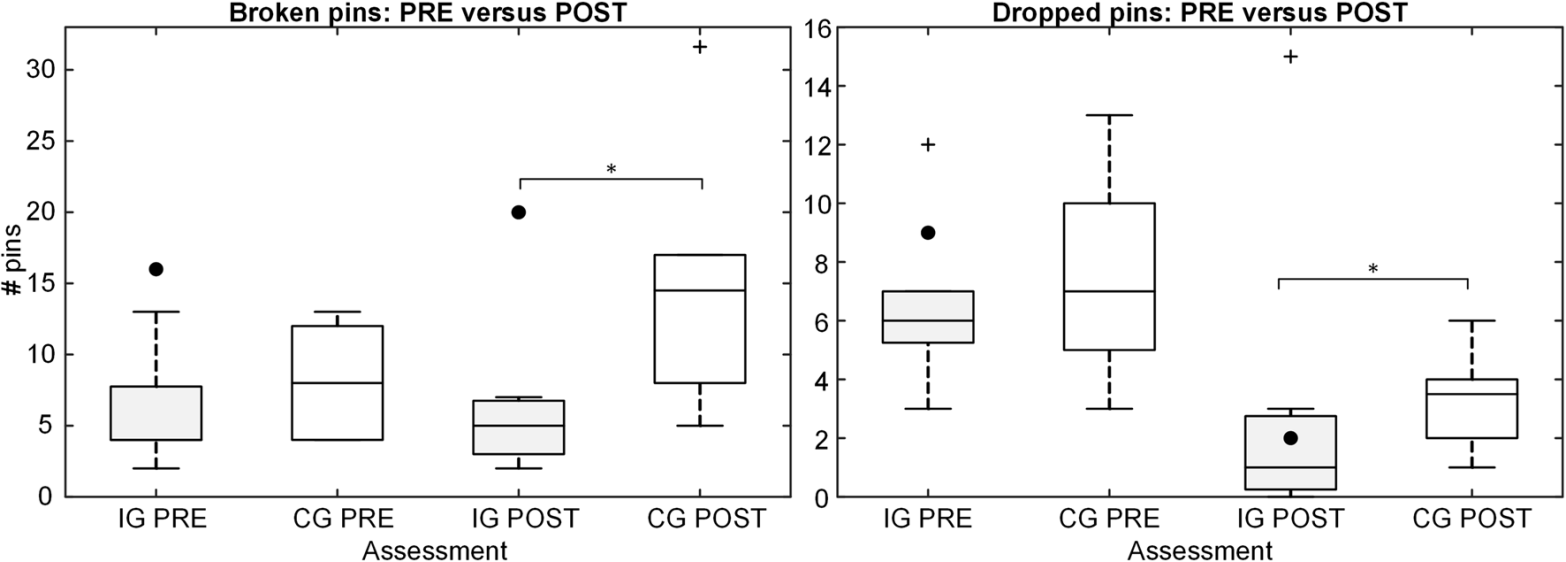
The number of broken (left) and dropped (right) pins in the pre- and posttest (real prosthesis) for the subjects in intervention and control groups (boxplots), and the participant with dysmelia (black dots). The boxplots depict median (horizontal line), interquartile range (box), min and max values (whiskers), and outliers (cross). The horizontal bars indicate a statistically significant difference (*, *p* < 0.05). IG and CG stand for intervention and control group, while PRE and POST refer to pre- and posttest, respectively

The distribution of the failed trials during pre- and posttest in IG and CG is shown in Fig. 10. The median number of broken pins did not change significantly between the two tests in either of the groups. Nevertheless, the subjects in the IG broke significantly (*p* < 0.05) fewer pins in the posttest compared to the CG (5/3.8 versus 14.5/9). The subjects in both groups dropped fewer pins in the posttest but the difference was significant only between IG and CG in the posttest (1/2.5 versus 3.5/2). The limb-deficient subject followed the trend of the other participants and dropped fewer pins after the training. However, she broke more pins in the posttest (16 versus 20).

Finally, the results of the post-questionnaire show that the IG rated the level of difficulty of the real prosthesis task significantly lower (*p* < 0.05) than the CG, namely 5.2 ± 1.9 versus 7.1 ± 0.9. This may indicate that the training with the AR system improved the subjects' skills to such an extent that they found the real prosthesis task significantly easier than the CG, which did not practice with the AR system. Moreover, the IG rated the fun factor and the associated motivation during the training sessions with the AR system with an average score of 8.7 ± 1.3. In addition, the visual feedback provided by the AR system was rated as very helpful (8.5 ± 1.7).

## Discussion

We presented a novel AR system that allows for practicing prosthesis control using pattern classification while interacting with the scene from a first-person perspective. The system was assessed by implementing a training protocol designed to stimulate the subjects to adjust wrist rotation and modulate grasping force. Moreover, we evaluated a transfer of skills to controlling a real prosthesis. The results have demonstrated that the developed framework was indeed effective: the training consistently improved the performance across sessions, and the performance in the task with the real prosthesis increased significantly only in the group that received the AR training, which also outperformed the control group in the posttest. In addition, the subjects reported positive user experience with the system, and they appreciated the provided visual force feedback. The main aim of this study was to evaluate the system by addressing its technical aspects, effectiveness, and user impression. The present study, however, cannot elucidate which component of the training protocol was particularly beneficial for the improvement. The subjects might have improved in the posttest because the AR environment allowed them to practice pattern classification control, closed-loop control with force feedback and/or wrist rotation via tilted bar (or even due to a combination of all these elements). Furthermore, the assessors were not blinded to the subjects’ group allocation during the pre- and posttests which could be a potential source of bias. Nevertheless, the goal of the present study was to develop and demonstrate a flexible training framework that in fact can be used to address such questions in the future.

Table 2 provides a comparison between the present solution and the similar systems presented in the literature. In general, only a few systems providing AR experience from the first-person perspective have been developed (all recently). The framework presented in this manuscript goes beyond the state of the art in several aspects: the force feedback is integrated into the AR, the field of view is substantially increased, the control is flexible and the system was evaluated systematically in a pool of subjects including an intervention and control group.

**Table 2:** Comparison to recent AR-based first-person perspective systems for myoelectric control

Study	Force feedback	Field of view (°)	Pattern recognition	Number of DOFs	Real prosthesis	Number of subjects (amputees)	Open Source
Nishino et al. [28]	No	23	No	2 + 0	Yes	5 (0)	No
Hunt et al. [29, 31]	No^a^	34	No^b^	–	No	3 (0)	No
Sharma et al. [32]	No^a^	34	Yes, LDA	2 + 2	Yes	3 (0)	No
Palermo et al. [33]	No	34	No^b^	2 + 2	No	5 (0)	No
Present work	Yes	110	Yes, LDA	3 + 2	Yes	14 (1)	Yes

^a^Grasping force information displayed on an external screen, not available to the subject in AR

^b^Pre-defined movements recognized by the Myo armband (e.g. “wave in”) mapped to virtual limb movements (e.g. wrist pronation/supination)

A particular advantage of the present AR system is that it offers flexibility in designing the training while providing immersion and a sense of realism due to first-person view and a mix of real and virtual elements. This might facilitate switching from the simulated to the real task since, for instance, during the training, the subjects interact with elements of the physical setup. In the present study, the flexibility of the framework has been demonstrated by imposing an additional demand for wrist rotation through the introduction of the virtual vertical bar that could be rotated, which is not possible in the real clothespin test. In addition, continuous and proportional feedback on grasping force was displayed to the subject to facilitate the learning of prosthesis control. Recent studies [40, 41] have emphasized that this is indeed an important role of explicit supplementary feedback. Importantly, the AR feedback was provided by the system only when needed, i.e., when the hand was in proximity to an object that can be interacted with or when the object was manipulated. Finally, the feedback was visually unobtrusive because it was dynamically rendered so that it never blocked the user’s view on relevant objects. The subjects reported that the force information was indeed useful. The pins were compliant and therefore the subjects could regulate the force by simply observing the pins; however, the direct visual assessment might not be that clear all the time (see Fig. 6c, d) and this might be the reason why additional explicit feedback was judged beneficial.

The subjects practiced with a limited number and range of grasping forces (Table 1) because the idea was to mimic the physical clothespin test. Nevertheless, we assume that this was enough for the subjects to learn the general principle of prosthesis force modulation, especially since the force feedback from the prosthesis was continuous. Therefore, the subjects could observe how their muscle activation was “transformed" into the grasping force while they modulated the contraction to reach a specific target window. The range of forces was in the lower half of the prosthesis capabilities but this also where the precise modulation is likely to be most relevant (handling more delicate objects). However, if and how well the subjects could generalize the learned grasping strategies to other force levels and tasks was not explicitly tested in the present study. Nevertheless, one of the important advantageous of the virtual framework is that force levels, ranges and training protocol can be easily and arbitrarily changed to address the training goals.

An important requirement for the presented system was low visual and control latency. The visual latency was dominated by acquiring stereo camera frames to RAM (ca. 50 ms) and hardware-accelerated drawing to the HMD screen (ca. 80 ms), resulting in a largely smooth image reproduction with low visual latency, which was not perceived as irritating, especially in the stationary task. Current related studies indicated significantly higher latencies for their systems, e.g. 500–800 ms [32]. The control latency was due to wireless EMG acquisition (ca. 150 ms), pattern recognition and movement controller update (ca. 70 ms), resulting in an average delay of under 250 ms. This is above an optimal delay in prosthesis control [42] and can be therefore further improved in future work. Nevertheless, the results showed that the delay did not negatively affect the transfer of skills nor the user experience.

Another important goal of the present system was the wide field of view. While the Microsoft HoloLens, which is often used in related works [31–33], has a diagonal field-of-view of 34° for the effective AR usable area, the HMD and stereo camera used in the present work offer a diagonal field-of-view of 110°. A larger field of view allows the subject to assess the scene without turning the head.

**Fig. 11:**
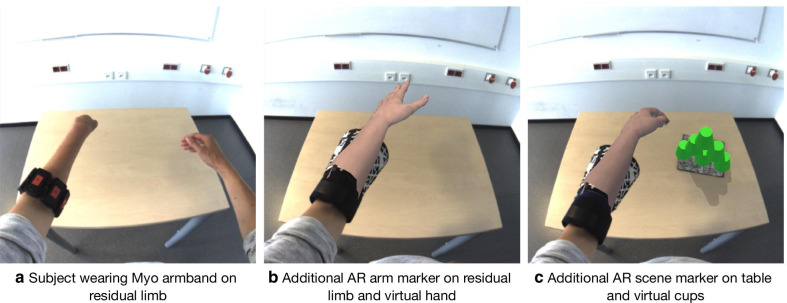
Modularity allows customization and implementation of the novel tasks. The scene from the subject perspective with no AR elements is shown on the left. The middle panel illustrates the addition of an AR arm marker and a virtual hand. The right panel depicts an AR scene marker and a setup of the cup-stacking task

The described solution combines diverse technologies into a complex system. The different components required careful adjustment to accomplish successful interplay. To enable further research, the source code and the design of our system along with extensive documentation and ready-to-use examples are released and freely available under the GPL3 license on the project’s website https://github.com/arlimb/arlimb. The modular design enables easy replacement of single components or integration into other projects. The hardware components can be easily combined, e.g. EMG sensors or AR HMDs. The usage of the widespread Unity 3D application environment enables high reusability and easy extension. The scenes, which were not tested explicitly in the present experiment, but are already included in the framework are Target Achievement Control (TAC) [16], Box and Blocks, Cup Stacking [39, 43] and Book turn tests. Figure 11 illustrates the extensibility of the framework using the cup-stacking test as an example. Figure 11a shows a first-person view of a test subject with dysmelia wearing the Myo armband. In Fig. 11b, the AR arm marker was added, and the virtual hand was drawn as an overlay in real-time. In Fig. 11c, a second marker was placed on the table to introduce a stack of cups to the scene. The subject could now control the virtual hand and interact with the cups.

The developed Unity 3D modules can be used for similar purposes or adapted for other experiments. Further scenarios can be easily created by taking advantage of the rich set of free and commercial assets available for the Unity 3D platform. In terms of mechanics, our system already comes with the detection of collision and grasping between the virtual hand and scene objects and includes visual elements for displaying grasp force and muscular activity. The latter could be used to implement EMG feedback, which can be an effective approach to close the loop for better online control and/or learning, as demonstrated recently [44–47].

The results of a meta-analysis [48] demonstrated that virtual reality rehabilitation programs are growing in popularity and are more effective for developing motor control than traditional programs. The author proposed that a combination of user excitement and physical and cognitive fidelity leads to improved outcomes. Upper limb prosthesis training often requires the repetition of well-defined tasks. Since Unity 3D is often used in a game development context, it is particularly suitable for the creation of gamified user experiences [11]. Gamification of test and training applications might be a way to increase patient motivation and concentration in experimental situations.

## Conclusions

The present manuscript describes a novel system for the training of pattern classification prosthesis control. To motivate and engage the subjects and facilitate the transfer of skills to controlling a real prosthesis, the system is based on augmented reality with the view from the first-person perspective. This approach provides immersion and realism. The training framework was systematically tested by implementing a training protocol with intervention and control groups including able-bodied participants and a subject with limb deficiency. The results demonstrated that the training was indeed effective leading to improvement in performance with virtual as well as real prosthesis (skill transfer). In addition, as assessed by a questionnaire, the overall user experience was positive and the subject appreciated the visual feedback on grasping force provided during the training. Therefore, the presented platform is a promising training instrument and the source code is available for download to facilitate further work and development.

## Supplementary Information

**Additional file 1.** Links and descriptions to a video demonstrating the system and to the project website.

## Data Availability

The data used in the current study are available from the corresponding author on reasonable request.

## Abbreviations

AR: Augmented reality
VR: Virtual reality
IC: Intervention group
CG: Control group
EMG: Electromyography
IMU: Inertial measurement unit
HMD: Head-mounted display
LDA: Linear discriminant analysis
SDK: Software development kit
TCP/IP: Transmission Control Protocol/Internet Protocol
DOF: Degree of freedom
