# Supplementary material for: Immersive augmented reality system for the training of pattern classification control with a myoelectric prosthesis
Source: J Neuroeng Rehabil. 2021 Feb 4;18:25. doi: 10.1186/s12984-021-00822-6 (PMC7860185; doi:10.1186/s12984-021-00822-6)
Supplement: Supplementary file 1 — Additional file 1. Links and descriptions to a video demonstrating the system and to the project website. [file 12984_2021_822_MOESM1_ESM.docx]

Supplementary material

## Demonstration video

Accompanying this article we have compiled a video in which the essential components of our system are presented. First, the general structure of the system is explained and a number of trials are shown as examples, in which the test subject with dysmelia controls the real prosthesis during pre- and post-testing. Then some trials are shown in which the test subject moves virtual clothespins with the virtual arm, both in side-view and from the test subject’s perspective simultaneously. The video is available on the main page of the project website <https://github.com/arlimb/arlimb>.

## Source code and documentation

To enable further research, the source code and the design of our system along with extensive documentation and ready-to-use examples are released and freely available under the GPL3 license on the project website <https://github.com/arlimb/arlimb>.
